# Supplementary material for: Metagenomic Sequencing of Positive Blood Culture Fluid for Accurate Bacterial and Fungal Species Identification: A Pilot Study
Source: Microorganisms. 2023 May 10;11(5):1259. doi: 10.3390/microorganisms11051259 (PMC10224492; doi:10.3390/microorganisms11051259)
Supplement: Supplementary file 1 [file microorganisms-11-01259-s001.zip › microorganisms-2353221-supplementary.pdf]

Supplemental Table S1. Detailed Conventional Microbiological Results.

UCLA ID #	Final ID By Conventional Methods	Blood Culture Stain Results	MALDI-TOF Performed?	MALDI-TOF ID	Other Micro Results or Morphological Description
UCLA_467	*Staphylococcus epidermidis*	Gram positive cocci in clusters	Yes	*S. epidermidis*	Coagulase tube test negative
UCLA_468	*Staphylococcus epidermidis*	Gram positive cocci in clusters	Yes	*S. epidermidis*	Coagulase tube test negative
UCLA_469	*Staphylococcus epidermidis*	Gram positive cocci in clusters	Yes	*S. epidermidis*	Coagulase tube test negative
UCLA_470	*Staphylococcus epidermidis*	Gram positive cocci in clusters	Yes	*S. epidermidis*	Coagulase tube test negative
UCLA_471	*Staphylococcus epidermidis*	Gram positive cocci in clusters	Yes	*S. epidermidis*	Coagulase tube test negative
UCLA_472	*Staphylococcus epidermidis*	Gram positive cocci in clusters	Yes	*S. epidermidis*	Coagulase tube test negative
UCLA_473	*Staphylococcus epidermidis*	Gram positive cocci in clusters	Yes	*S. epidermidis*	Coagulase tube test negative
UCLA_478	*Slackia exigua*	Gram positive coccobacilli	Yes	*Slackia exigua*	Anaerobic growth
UCLA_496	*Candida tropicalis*	Yeast	Yes	*Candida tropicalis*	
UCLA_497	*Candida glabrata*	Yeast	Yes	*Candida glabrata*	
UCLA_498	*Fusarium proliferatum*	Fungal element	Yes	*Fusarium proliferatum*	
UCLA_499	*Desulfovibrio* spp.	Gram negative rod	Yes	*Desulvibrio spp.*	Anaerobic growth
UCLA_500	*Candida lusitaniae*	Yeast	Yes	*Candida lusitaniae*	
UCLA_501	*Bacteroides dorei*	Gram negative rod	Yes	*Bacteroides dorei*	Anaerobic growth
UCLA_502	*Candida albicans*	Yeast	Yes	*Candida albicans*	
UCLA_503	*Bacteroides thetaiotaomicron*	Gram negative rod	Yes	*Bacteroides thetaiotaomicron*	Anaerobic growth
UCLA_505	*Yellow pigmented rapid growing Mycobacterium spp*	Gram positive beaded rod; AFB positive	Yes	No ID	Yellow pigmented rapid grower
UCLA_510	*Proteus mirabilis*	Gram negative Rod	Yes	*Proteus mirabilis*	
UCLA_517	*Escherichia coli*	Gram negative Rod	Yes	*E. coli*	Lactose positive
UCLA_518	*Staphylococcus lugdunensis*	Gram positive cocci in clusters	Yes	*Staphylococcus lugdunensis*	
UCLA_519	*Streptococcus anginosus group*	Gram positive cocci in chains	Yes	*Streptococcus Intermedius*	
UCLA_520	*Clostridium butyricum*	Gram positive cocci in chains	Yes	*Clostridium butyricum*	
UCLA_524	*Mycobacterium avium complex*	Gram positive beaded rod; AFB positive	No	NA	MAC DNA probe positive
UCLA_558	*Staphylococcus epidermidis*	Gram positive cocci in clusters	Yes	*S. epidermidis*	Coagulase tube test negative
UCLA_559	*Enterococcus faecium*	Gram-positive cocci in pairs and chains	Yes	*Enterococcus faecium*	
UCLA_560	*Fusobacterium nucleatum*	Gram-negative rod	Yes	*Fusobacterium nucleatum*	
UCLA_561	*Staphylococcus haemolyticus*	Gram-positive cocci in clusters	Yes	*Staphylococcus haemolyticus*	
UCLA_562	*Staphylococcus epidermidis*	Gram positive cocci in clusters	Yes	*S. epidermidis*	Coagulase tube test negative
UCLA_596	*Aspergillus flavus-oryzae* group	Fungal element	Yes	*Aspergillus flavus-*oryzae group	Fungus balls were seen inside the blood culture bottle
UCLA_1007	*Mycobacterium avium complex*	Gram positive beaded rod; AFB positive	No	NA	MAC DNA probe positive
